# High-dimensional multinomial multiclass severity scoring of COVID-19 pneumonia using CT radiomics features and machine learning algorithms

**DOI:** 10.1038/s41598-022-18994-z

**Published:** 2022-09-01

**Authors:** Isaac Shiri, Shayan Mostafaei, Atlas Haddadi Avval, Yazdan Salimi, Amirhossein Sanaat, Azadeh Akhavanallaf, Hossein Arabi, Arman Rahmim, Habib Zaidi

**Affiliations:** 1 Division of Nuclear Medicine and Molecular Imaging, Geneva University Hospital, 1211 Geneva, Switzerland; 2 Division of Clinical Geriatrics, Department of Neurobiology, Care Sciences and Society, Karolinska Institutet, Stockholm, Sweden; 3 School of Medicine, Mashhad University of Medical Sciences, Mashhad, Iran; 4 Departments of Radiology and Physics, University of British Columbia, Vancouver, BC Canada; 5 Department of Integrative Oncology, BC Cancer Research Institute, Vancouver, BC Canada; 6 Geneva University Neurocenter, Geneva University, Geneva, Switzerland; 7 Department of Nuclear Medicine and Molecular Imaging, University of Groningen, University Medical Center Groningen, Groningen, The Netherlands; 8 Department of Nuclear Medicine, University of Southern Denmark, Odense, Denmark

**Keywords:** Computational biology and bioinformatics, Medical research

## Abstract

We aimed to construct a prediction model based on computed tomography (CT) radiomics features to classify COVID-19 patients into severe-, moderate-, mild-, and non-pneumonic. A total of 1110 patients were studied from a publicly available dataset with 4-class severity scoring performed by a radiologist (based on CT images and clinical features). The entire lungs were segmented and followed by resizing, bin discretization and radiomic features extraction. We utilized two feature selection algorithms, namely bagging random forest (BRF) and multivariate adaptive regression splines (MARS), each coupled to a classifier, namely multinomial logistic regression (MLR), to construct multiclass classification models. The dataset was divided into 50% (555 samples), 20% (223 samples), and 30% (332 samples) for training, validation, and untouched test datasets, respectively. Subsequently, nested cross-validation was performed on train/validation to select the features and tune the models. All predictive power indices were reported based on the testing set. The performance of multi-class models was assessed using precision, recall, F1-score, and accuracy based on the 4 × 4 confusion matrices. In addition, the areas under the receiver operating characteristic curves (AUCs) for multi-class classifications were calculated and compared for both models. Using BRF, 23 radiomic features were selected, 11 from first-order, 9 from GLCM, 1 GLRLM, 1 from GLDM, and 1 from shape. Ten features were selected using the MARS algorithm, namely 3 from first-order, 1 from GLDM, 1 from GLRLM, 1 from GLSZM, 1 from shape, and 3 from GLCM features. The mean absolute deviation, skewness, and variance from first-order and flatness from shape, and cluster prominence from GLCM features and Gray Level Non Uniformity Normalize from GLRLM were selected by both BRF and MARS algorithms. All selected features by BRF or MARS were significantly associated with four-class outcomes as assessed within MLR (All *p *values < 0.05). BRF + MLR and MARS + MLR resulted in pseudo-R^2^ prediction performances of 0.305 and 0.253, respectively. Meanwhile, there was a significant difference between the feature selection models when using a likelihood ratio test (*p *value = 0.046). Based on confusion matrices for BRF + MLR and MARS + MLR algorithms, the precision was 0.856 and 0.728, the recall was 0.852 and 0.722, whereas the accuracy was 0.921 and 0.861, respectively. AUCs (95% CI) for multi-class classification were 0.846 (0.805–0.887) and 0.807 (0.752–0.861) for BRF + MLR and MARS + MLR algorithms, respectively. Our models based on the utilization of radiomic features, coupled with machine learning were able to accurately classify patients according to the severity of pneumonia, thus highlighting the potential of this emerging paradigm in the prognostication and management of COVID-19 patients.

## Introduction

The highly contagious SARS-CoV-2 virus has led to significant morbidity and mortality worldwide^1^. Pneumonia is regarded as one of the main complications of COVID-19 disease, which can lead to lethal conditions while escalating the cost of healthcare^2^. The most popular diagnostic test considered as the gold standard for coronavirus disease is the reverse transcription polymerase chain reaction (RT-PCR) assay^3^. While highly specific, RT-PCR has shown low sensitivity, as studies have reported significant false-negatives in patients who had abnormalities in their chest CT images confirmed with secondary follow-up RT-PCR to be positive for COVID-19^4^.

CT aids in the diagnosis and management of COVID-19 patients and could be potentially used as an outcome/survival prediction tool, towards enhanced treatment planning^5^. CT scanning has been utilized as a highly sensitive tool for COVID-19 diagnosis^6^ since it is fast and generates quantifiable features (e.g., the extent to which lung lobes are involved) and non-quantifiable features (e.g., ground-glass opacities and their laterality) to assess COVID-19 pneumonia, besides the enhanced sensitivity compared to RT-PCR^7^.

Severity can be defined as an index that depicts the effects of a disease on mortality, morbidity, and comorbidities and has the potential to help physicians manage the patients more decently whether in patients with cancer or with non-cancer diseases^8,9^. A number of severity scoring systems have been proposed to quantify disease advancement in patients, including general assessments (e.g., APACHE score) and disease-specific ones (e.g., Child–Pugh score)^10^. Several conventional scoring systems have been proposed for COVID-19 severity assessment^11^. These include the usage of patient clinical, comorbidity, and laboratory data, which are all helpful in constructing predictive models for severity assessment in COVID-19^12^.

There has also been a growing interest in using imaging data of patients, such as thoracic CT images. For example, a study by Sanders et al.^13^ computed the score of CT images in patients with cystic fibrosis and evaluated the prognostic ability. A promising line of research that emerged recently reported on the CT severity index and its correlation with acute pancreatitis severity^14–16^. The COVID-19 Reporting and Data System (CO-RADS) was suggested for standardized visual assessment of COVID-19 pneumonia to enhance agreement between radiologists^17^. This system includes features for the diagnosis of COVID-19 and consists of a 6-point scale for categorizing patient CT images. In addition, other guidelines aiming to reach consensus when interpreting COVID-19 suspected chest CT images were proposed^18^. These guidelines are mostly based on visual assessment of images; e.g. the amount to which lung lobes are involved, the volume of which is infected, and anatomical assessments.

Francone et al.^19^ reported a study on the correlation between CT score and the severity of coronavirus disease. Zhao et al.^20^ also conducted research on the measurement of the extent to which lung lobes are infected and evaluation in COVID-19 patients' prognosis. Li et al.^21^ also confirmed the association between chest CT score and COVID-19 pneumonia severity. At the same time, most scoring systems involve visual assessment and hence are time-consuming^20,21^. In this regard, medical image analysis using machine learning (ML) and radiomics has been applied to quantify features to tackle these main challenges^22^.

The field of radiomics opens pathways for the study of normal tissues, cancer, cardiac disease, and many other diseases, including potentially the newly emerging COVID-19 disease^23–30^. Specifically, Xie et al.^31^ evaluated the potential of a radiomics framework to diagnose COVID-19 from CT images. Di et al.^32^ also studied whether radiomics features can help to distinguish between pneumonia of COVID-19 and that of other viral/bacterial causes. A number of studies reported on the application of radiomics analysis to CT images towards COVID-19 classification and prognostication^33–36^. Homayounieh et al.^37^ assessed the prognostic power of CT-based radiomics features to determine severe and non-severe cases. In another study, Li et al.^38^ proposed a radiomics model based on CT images and classified patients based on the criticality of their disease. A recent study by Yip et al.^39^ applied a robust radiomics model to CT images to predict the severity of COVID-19 disease in patients. All above models pursued binary task performance, which reduced multiclass classification to two class approaches. However, in the real clinical triage situation, scoring systems consist of multi-class datasets. In the present study, involving a large cohort of patients, we aimed to construct a CT radiomics-based multi-class classification model to predict the severity of COVID-19 pneumonia.

## Materials and methods

### Data description

**Figure 1:**
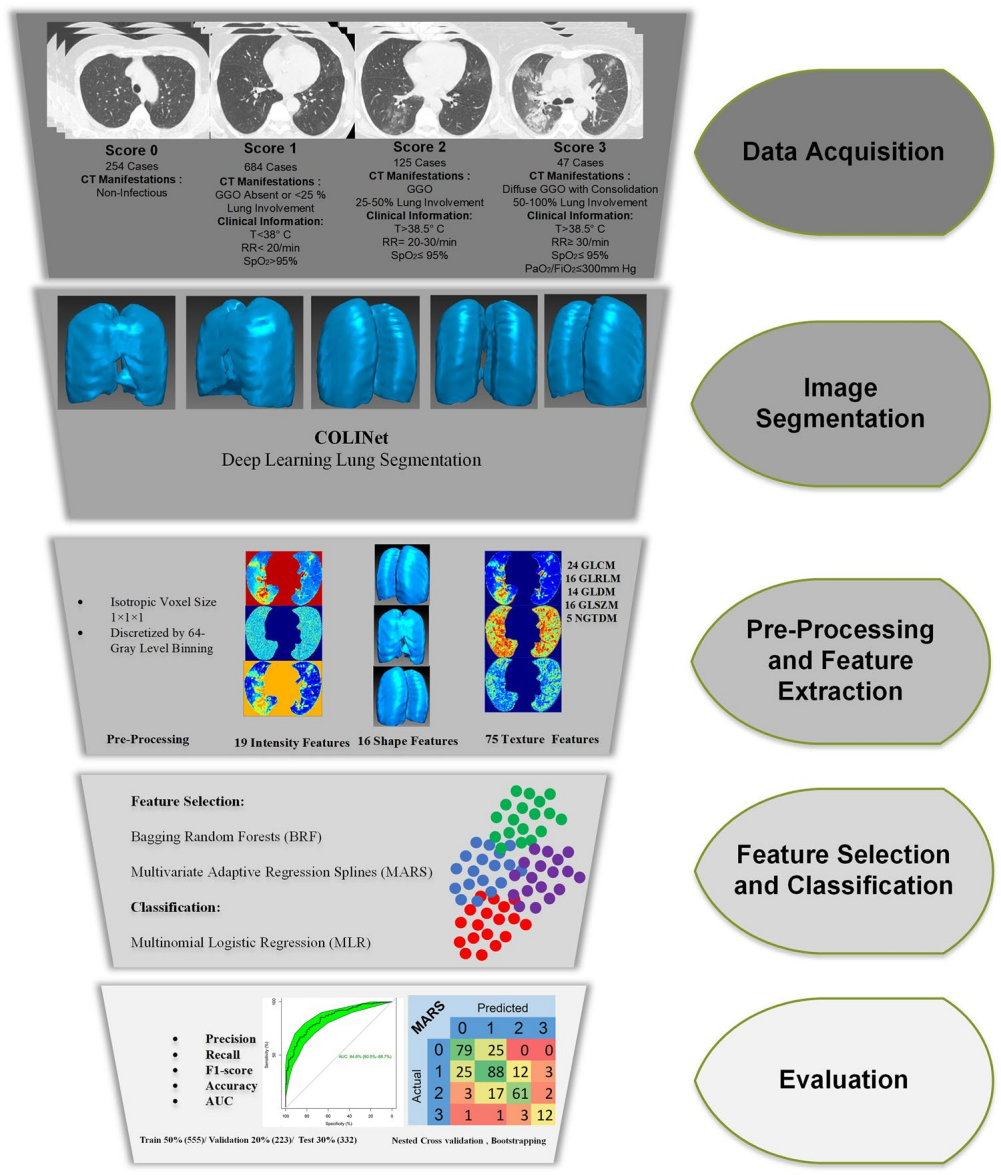
Different steps of the current study, including data acquisition, image segmentation using COLI-Net, image preprocessing and feature extraction, machine learning and evaluation method and metrics. GGO: ground glass opacities, T: Temperature, RR: Respiratory Rate, SpO_2_: Peripheral Capillary Oxygen Saturation, PaO_2_: Partial Pressure of Oxygen. FiO_2_ = Fraction of Inspired Oxygen.

Figure 1 presents the different steps performed in this study. All experiments were performed in accordance with relevant guidelines and regulations.

### Datasets and segmentation

**Figure 2:**
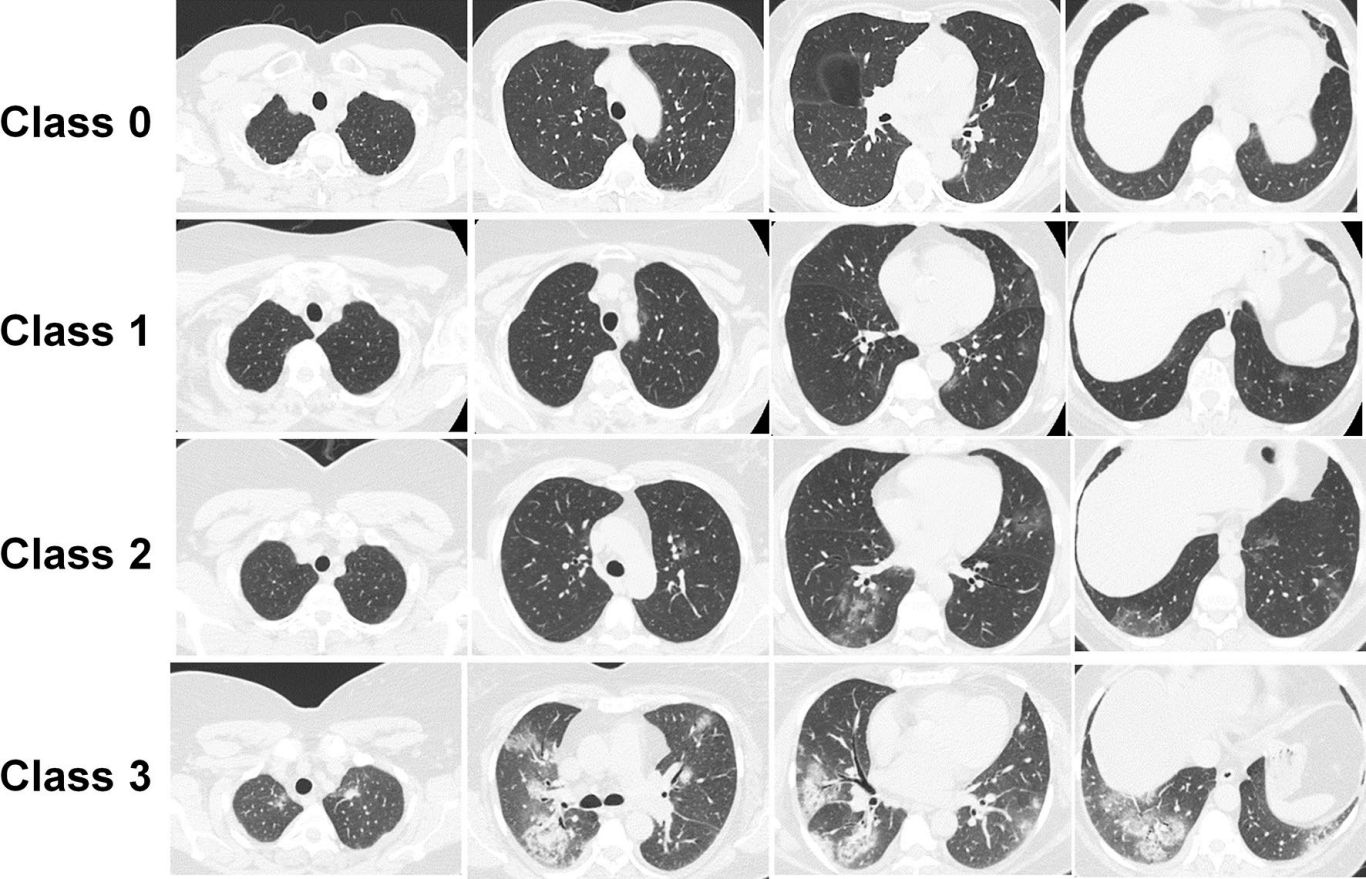
Examples of patient CT images belonging to different classes with different scores.

This study is based on the MosMed Dataset^40^ consisting of 1110 patient CT scans, also utilized in other efforts^39,41^. Ethics approval and consent to participate were not needed since the study was preformed on open access online dataset. In the class zero, the patient has neither clinical symptoms (e.g. fever) nor CT findings in favor of any kind of pneumonia (Class 0, non-pneumonic)^40^. The 1st class contains patients who have a low-temperature fever (t < 38 °C) in addition to a mild increase in respiratory rate (RR < 20) while showing none or < 25% ground-glass opacity (GGO) involvement (Class 1, COVID-19 with mild severity)^40^. Patients in the 2nd class have a higher body temperature (t > 38.5 °C) with a RR of 20–30, while CT scan shows 25–50% involvement of lung parenchyma (Class 2, COVID-19 with moderate severity)^40^. Patients in the 3rd class have high body temperature and RR of 30 or more, with CT findings of 50% to diffuse involvement in addition to organ failure and shock signs (Class 3, severe COVID-19)^40^. Each of the classes, namely 0, 1, 2, and 3, included 254, 684, 125, and 47 patients, respectively^40^. The median age was 47 (ranging from 18 to 97), and 42% of patients were female. Figure 2 shows an example of representative CT images for each class^40^.

All CT images were automatically segmented using a deep learning-based algorithm for whole lung segmentation^42^. After whole-lung 3D segmentation, all images were reviewed and modified to ensure correct 3D-volume lung segmentation.

### Image preprocessing and feature extraction

To preserve image resolution and efficient radiomics feature extraction, all images were cropped to lung region and then resized to 296 × 216 matrix size^33,43^. Subsequently, image voxels were resized to an isotropic voxel size of 1 × 1 × 1 mm^3^ (for invariant texture feature extraction) and image intensity were discretized to 64-binning size^44^. The extracted features from the whole-lung segmented regions, totalling 110, included shape (n = 16), intensity (n = 19), and texture features, namely second-order texture of gray-level co-occurrence matrix (GLCM, n = 24), and high-order features, namely gray-level size-zone matrix (GLSZM, n = 16), neighbouring gray tone difference matrix (NGTDM, n = 5), gray-level run-length matrix (GLRLM, n = 16) and gray-level dependence matrix (GLDM, n = 14). Radiomics feature extraction was performed using the Pyradiomics Python library^45^, which is compliant with the image biomarker standardization initiative (IBSI)^44^. In addition, feature maps were generated using voxelwise feature extraction.

### Feature selection and classification and evaluation

In this study, we used two different feature selection algorithms, including Bagging Random Forests (BRF) and Multivariate Adaptive Regression Splines (MARS)^46^. BRF and MARS algorithms were implemented in "VSURF" and "earth" R packages, respectively. Importance values (IVs) were calculated using generalized cross-validation criterion with normalization. For multiclass classification, we implemented multinomial logistic regression (MLR) using the "mnlogit" R package. The MLR model fitness indices included *p *value of the Wald test (corrected for false-discovery rate via Benjamini and Hochberg method), pseudo R^2^, as well as Akaike information criterion (AIC, goodness of fit indices in generalized linear regression models). In the MLR model, class 0 served as a reference class whereas statistical comparison between two predictive models was performed by the Likelihood Ratio Test.

The dataset was divided into 50% (555 samples), 20% (223 samples), and 30% (332 samples) as training, validation, and untouched test datasets, respectively. The nested fivefold cross-validation with grid search was used to validate models and estimate tuning hyper-parameters based on the minimization of GCV error rate. In our nested fivefold cross-validation processing, there were 5 outer folds (i.e., training and testing sets) and 5 inner folds (i.e., training and validation sets) where the total number of trained models was 25 for each classifier. We report mean precision, recall, F1-score, and accuracy and their standard deviation (SD) for different classes in each model based on the 30% untouched test set with bootstrapping (n = 1000) to ensure reproducibility. In addition, the areas under the receiver operating characteristic (ROC) curve (AUCs) for multi-class classification models were calculated and compared for both models using “multiROC” and “pROC” R packages, respectively.

## Results

**Figure 3:**
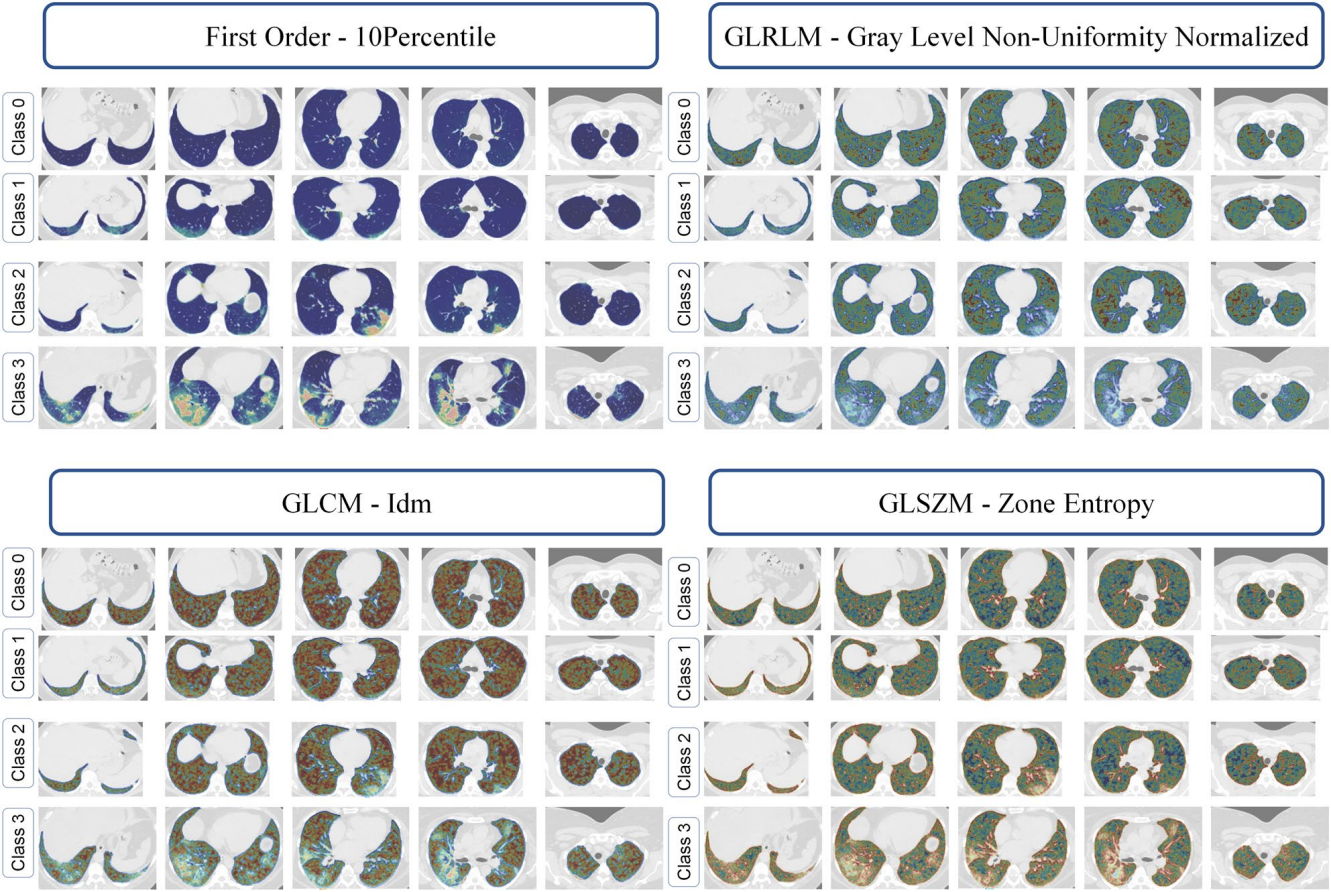
Examples of selected features (10Precentile from first order, Gray level Non-Uniformity Normalized from GLRLM, Idm from GLCM and Zone Entropy from GLSZM) in different class cases and different slices.

Table 1 summarizes the selected features and their relative importance value by BRF and MARS for multiclass classification. These features were selected in train/validation sets using nested cross validation and grid searches. Twenty-three radiomic features were selected by BRF, including 11 from first-order, 9 from GLCM, 1 from GLRLM, 1 from GLDM, and 1 from shape features. Among these features, Correlation (IV: 100%) and Cluster Tendency (IV: 88%) from GLCM, Mean Absolute Deviation (IV: 80%), Robust Mean Absolute Deviation (IV: 72%) and variance (IV: 70%) from first-order features were selected as the most important ones. In the MARS algorithm, 10 features were selected with high IVs, including 2 from first-order, 1 from GLDM, and 1 from GLCM. The highest IV was achieved by mean absolute deviation (IV: 100%) and skewness (IV: 55%) from first-order, Gray Level Variance from GLDM (IV: 53%), and Correlation from GLCM (IV: 54%). The mean absolute deviation, skewness, variance from first-order, flatness from shape, cluster prominence from GLCM features, and Gray Level Non Uniformity Normalize from GLRLM were selected by both BRF and MARS algorithms. Figure 3 depicts the feature map of different radiomics features in different classes (10Precentile from first order, Gray level Non-Uniformity Normalized from GLRLM, Idm from GLCM and Zone Entropy from GLSZM).

**Table 1:** Selected features by Bagging Random Forests (“VSURF” R package) and multivariate adaptive regression splines (“earth” R package) for multi-class classification using nested fivefold cross validation based on the training set (50% of the samples, N = 555) and the validation set (20% of the samples, N = 223).

Algorithm	Selected variables	Feature type	Relative importance value (%)
Bagging Random Forests	First Order	Mean Absolute Deviation	80
	First Order	Robust Mean Absolute Deviation	72
	First Order	Variance	70
	First Order	Interquartile Range	68
	First Order	Kurtosis	62
	First Order	Skewness	61
	First Order	Entropy	42
	First Order	10Percentile	40
	First Order	90Percentile	36
	First Order	Energy	30
	First Order	Mean	20
	GLCM	Correlation	100
	GLCM	Cluster Tendency	88
	GLCM	Sum Squares	66
	GLCM	Inverse Variance	60
	GLCM	Cluster Shade	55
	GLCM	Cluster Prominence	54
	GLCM	Joint Entropy	52
	GLCM	Idm	48
	GLCM	Id	44
	GLDM	Dependence Variance	65
	GLRLM	Gray Level Non Uniformity Normalize	51
	Shape	Flatness	18
Multivariate Adaptive Regression Splines	First Order	Mean Absolute Deviation	100
	First Order	Skewness	55
	First Order	Variance	11
	GLCM	Correlation	54
	GLCM	Cluster Prominence	47
	GLCM	Difference Entropy	36
	GLDM	Gray Level Variance	53
	GLRLM	Gray Level Non Uniformity Normalize	10
	GLSZM	Zone Entropy	20
	Shape	Flatness	48

Relative importance value calculated using generalized cross-validation (GCV) criterion with normalization.

Table 2 summarizes the adjusted *p *value (by Benjamini and Hochberg method) of the Wald test and AIC for both feature selection algorithms using MLR model. All selected features yielded a significant *p *value (< 0.05). BRF + MLR and MARS + MLR resulted in pseudo R^2^ values of 0.305 and 0.253, respectively. However, there were significant differences between both predictive models when using a likelihood ratio test (*p *value = 0.046).

**Table 2:** Multinomial logistic regression for the selected features by “mnlogit” R package and the model’s fitness indices based on the testing set (N = 332).

Algorithm	Feature type		Adj. *p *value	Pseudo R^2^	AIC
Bagging Random Forests	First Order	Mean Absolute Deviation	< 0.001	0.305	782.6
	First Order	Robust Mean Absolute Deviation	< 0.001		
	First Order	Variance	< 0.001		
	First Order	Interquartile Range	< 0.001		
	First Order	Kurtosis	< 0.001		
	First Order	Skewness	< 0.001		
	First Order	Entropy	0.001		
	First Order	10Percentile	0.002		
	First Order	90Percentile	0.001		
	First Order	Energy	0.005		
	First Order	Mean	0.025		
	GLCM	Correlation	< 0.001		
	GLCM	Cluster Tendency	< 0.001		
	GLCM	Sum Squares	< 0.001		
	GLCM	Inverse Variance	< 0.001		
	GLCM	Cluster Shade	< 0.001		
	GLCM	Cluster Prominence	< 0.001		
	GLCM	Joint Entropy	< 0.001		
	GLCM	Id	0.001		
	GLCM	Idm	0.001		
	GLDM	Dependence Variance	< 0.001		
	GLRLM	Gray Level Non-Uniformity Normalize	0.009		
	Shape	Flatness	< 0.001		
Multivariate Adaptive Regression Splines	First Order	Mean Absolute Deviation	< 0.001	0.253	972.8
	First Order	Skewness	< 0.001		
	First Order	Variance	< 0.001		
	GLCM	Cluster Prominence	< 0.001		
	GLCM	Correlation	< 0.001		
	GLCM	Difference Entropy	< 0.001		
	GLDM	Gray Level Variance	< 0.001		
	GLRLM	Gray Level Non-Uniformity Normalize	< 0.001		
	GLSZM	Zone Entropy	< 0.001		
	Shape	Flatness	< 0.001		

*p *value by Wald chi-square test, Adj. *p *value: *P *value adjusted by Benjamini and Hochberg method, statistical comparison between two models showed non-significant difference by Likelihood Ratio Test: *P *value = 0.046, AIC: Akaike information criterion.

**Figure 4:**
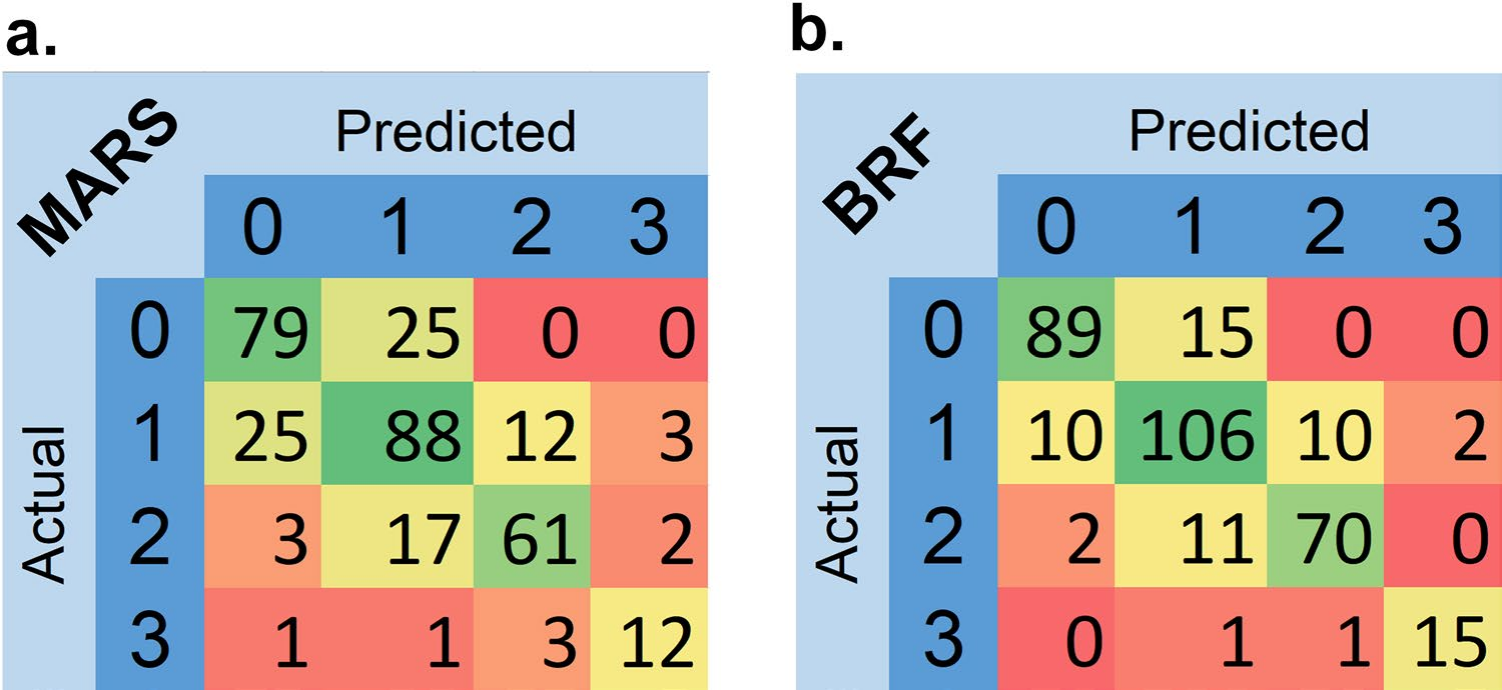
Four-by-four confusion matrix for (**a**) Multivariate Adaptive Regression Splines (MARS) and Bagging Random Forests (BRF).

**Figure 5:**
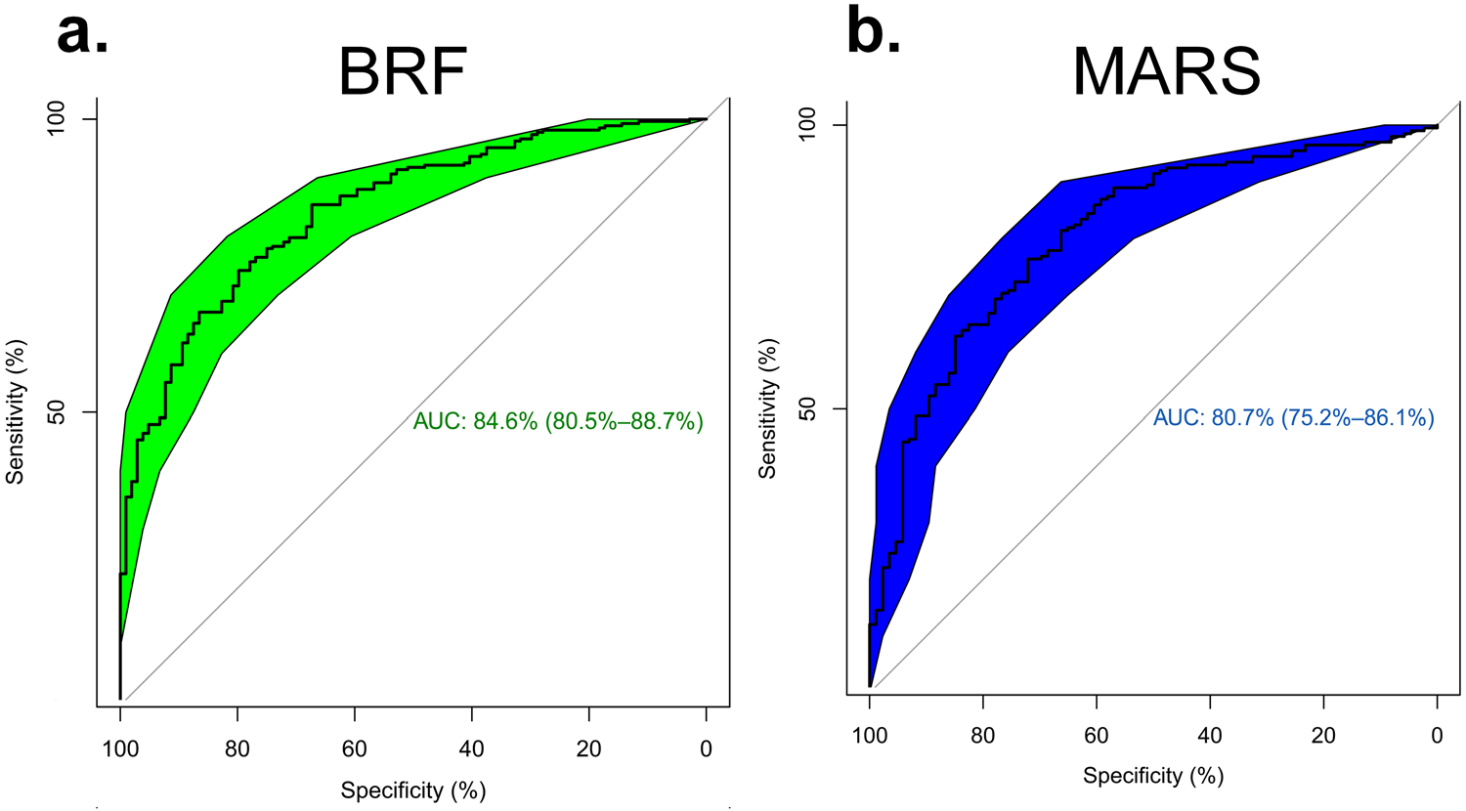
(**a**) ROC curve for assessing power of multi-class classification of the selected features in Bagging Random Forests (AUC = 0.846), and (**b**) Multivariate Adaptive Regression Splines (AUC = 0.807). Statistical comparison of ROC curves by “pROC” R package indicated significant difference (Z = 3.834, *p *value < 0.001).

Table 3 summarizes classification power indices, including mean (SD) Precision, Recall, F1-score, Accuracy, and AUC via multinomial logistic regression with 1000 bootstrapping samples for each model in untouched test dataset. In terms of F1-score, four-class mean F1-scores were 0.854 and 0.724 for BRF + MLR and MARS + MLR algorithms, respectively. The mean precision was 0.856 and 0.728, whereas the mean recall was 0.852 and 0.722 for BRF + MLR and MARS + MLR algorithms, respectively. BRF + MLR and MARS + MLR algorithms achieved an accuracy of 0.921 and 0.861, respectively, in four-class classification. AUCs (95% CI) for multi-class classification were 0.846 (0.805–0.887) and 0.807 (0.752–0.861) for BRF + MLR and MARS + MLR algorithms, respectively. According to the results of the classification metrics, the predictive power of the BRF + MLR model is higher than MARS + MLR. Figure 4 depicts the confusion matrices for both predictive models based on the testing set whereas Fig. 5 shows the ROC curves for our four-class classification methods.

**Table 3:** The classification power indices (SD) based on the testing set (N = 332) with 1000 bootstrapping samples based on the feature selection methods.

Algorithm	Class	Precision	Recall	F1-score	Accuracy	AUC (95% CI)
Bagging Random Forests	Class 1	0.881 (0.098)	0.855 (0.085)	0.868 (0.079)	0.918 (0.109)	0.846 (0.805–0.887)
	Class 2	0.800 (0.039)	0.828 (0.037)	0.812 (0.019)	0.852 (0.049)	
	Class 3	0.864 (0.105)	0.843 (0.079)	0.853 (0.096)	0.928 (0.117)	
	Class 4	0.882 (0.103)	0.882 (0.088)	0.882 (0.109)	0.988 (0.119)	
	Average/total	0.856	0.852	0.854	0.921	
Multivariate Adaptive Regression Splines	Class 1	0.731 (0.099)	0.760 (0.101)	0.745 (0.089)	0.837 (0.116)	0.807 (0.752–0.861)
	Class 2	0.671 (0.039)	0.688 (0.033)	0.679 (0.026)	0.750 (0.031)	
	Class 3	0.802 (0.119)	0.734 (0.101)	0.767 (0.098)	0.888 (0.121)	
	Class 4	0.706 (0.109)	0.706 (0.109)	0.706 (0.109)	0.970 (0.136)	
	Average/total	0.728	0.722	0.724	0.861

## Discussion

In the current study, we constructed a CT radiomics-based model to predict the severity of COVID-19 patients in a large cohort of patients. To this end, we extracted radiomics features from whole lung segmentations and selected high-importance features utilizing two different algorithms, namely BRF and MARS. The selected features were then fed to a multinomial logistic regression classifier for multiclass severity scoring. We achieved 0.846 (0.805–0.887) and 0.807 (0.752–0.861) for AUC, and 0.921 and 0.861 for accuracy in BRF- and MARS-selected features, respectively. We used an automatic model to segment chest CT images for two reasons. First, most CT scans performed in the COVID-19 pandemic era are low-dose. In addition, these scans are acquired with a high pitch. Hence, it is difficult for radiologists to find and follow lung fissures to manually detect or segment the anatomical lobes. As such, we used our previously constructed and validated deep learning model to fully segment the entire lung of each patient^33,34,42,43,47^.

Yip et al*.*^39^ conducted a study on the same dataset utilized in this work, aiming to evaluate some radiomics features towards severity class prediction in patients. They included all 1110 patient CT scans and extracted 107 radiomics features. The maximum relevance minimum redundancy (MRMR) and recursive feature elimination (RFE) algorithms were exploited for feature selection and analysis of the selected features using univariate and multivariate approaches using a logistic regression model to classify as accurately as possible. In their study, the patients were categorized into three severity categories, namely mild, moderate, and severe, to perform two-class classification tasks (mild vs. severe and moderate vs. severe) by splitting the data into training (60%) and test (40%) sets. The authors obtained an AUC of 0.65 in differentiating between moderate and severe cases, while their model performed better (AUC = 0.85) in distinguishing mild vs. severe forms of COVID-19 disease. In this work, we reached an overall AUC of 0.846. In our study and the one by Yip et al.^39^, feature extractions were performed using Pyradiomics^45^ as applied to the entire lung. Interestingly, there were some commonly selected features arrived at via feature selection in both studies, including Mean Absolute Deviation, 10Percentile, 90Percentile, and Mean from first order and Correlation from GLCM. These selected features in both studies could potentially be used as predictors as they provide information about the intensity and heterogeneity of the lung in COVID-19 patients.

A noticeable advantage of the study by Yip et al*.*^39^ was the use of a second radiologist observer who classified patients’ images into mild, moderate, and severe classes without paying attention to the default classification of the dataset provider. This method helped to observe the prediction power of the models in both “provider” and “radiologist” datasets. In addition, the study by Yip et al*.*^39^ may have reduced generalizability as it only predicts mild versus severe, and moderate versus severe disease, having reduced multiclass classification into two-class approaches. In the real clinical triage situation, the radiologist may benefit from a multiclass classification scheme for enhanced patient management, as provided by our study.

Multi-class classification is a difficult machine learning task^48^. Different studies have shown that ML/DL algorithms are capable of predicting much more decently when classifying binary categories, compared to multiple categories. For example, a study by Senan et al.^49^ showed that a specific DL network achieved an accuracy of 99% and AUC of 97.5% for binary classification (COVID-19 vs healthy) compared to an accuracy and AUC of 95% and 97.1%, respectively, for classifying CXRs into COVID-19, viral pneumonia, lung opacity, and healthy individuals.

Regarding multi-class classification studies on COVID-19, some studies showed promising results^50–52^. For instance, Wu et al.^53^ and Qian et al.^54^ evaluated the power of CXR-based and CT-based CNN models for differentiating between multiple classes of patients, including COVID-19, viral pneumonia, bacterial pneumonia, and healthy individuals, respectively. In addition to CNN models, some studies investigated multi-class categorization power of ML models. For example, Hussain et al.^55^ assessed COVID-19, bacterial, viral, and healthy CXRs using extracted features and five ML algorithms. These algorithms classified each CXR into one of the four aforementioned CXR categories. They reached an accuracy and AUC of 0.79 and 0.87, respectively. A study by Khan et al.^56^ evaluated CT-based ML algorithms, such as multi-class SVM. In a recent study by Moradi Khaniabadi et al.^34^, two-step ML algorithms were proposed for diagnosis and severity scoring from COVID-19 CT images. They performed three-class classification for two different diagnostic tasks (normal, other pneumonia, and COVID-19 pneumonia) and severity scoring (mild, moderate and severe). They extracted radiomic features form whole lungs and used multiple machine learning algorithms for feature selection and classification purposes. They reported 0.909 ± 0.026, 0.907 ± 0.056, and 0.982 ± 0.010 for precision, recall, and AUC for diagnostic purposes and 0.868 ± 0.123 precision, 0.865 ± 0.121 recall, and 0.969 ± 0.022 AUC for severity scoring using a random forest algorithm.

Homayounieh et al*.*^57^ included 315 patients in their study and extracted CT-based radiomics features from the lung to show that radiomics can predict patients’ outcome (inpatient vs. outpatient management) with an AUC of 0.84 while the radiologist assessment alone achieved an AUC of 0.69. Feature extraction was performed by applying the different preprocessing algorithms on images, with classification performed using logistic regression. They reported that adding clinical variables to the radiomics model can notably improve the predictability of a model for patient outcome prediction (AUC improved from 0.75 to 0.84). Another study conducted by Wei et al*.*^58^ evaluated the predictive ability of two models (one CT texture-based and one clinical) for determining the severity of each of the 81 COVID-19 patients. They showed that CT texture features could modestly predict whether the patient has common COVID-19 pneumonia or a severe one with an AUC of 0.93, which is comparable to that of the clinical-only model (AUC = 0.95). They also observed that several texture features had a moderate correlation with the clinical variables of patients.

Chaganti et al*.*^59^ studied Ground Glass Opacity (GGO) and consolidations that appear on a CT image of COVID-19 patients in an attempt to propose an automated method for segmenting and quantifying COVID-19 lesions. Their proposed method calculated the percentage of opacity and lung severity score using deep learning algorithms and was able to predict the severity with a decent performance. However, Chaganti et al.^59^ proposed a method trained only on the mentioned abnormalities and had a limited performance in other abnormalities quantification. Even with improving segmentation algorithms, this method would be limited because of the highly heterogeneous nature of COVID-19 pneumonia in addition to ignoring the shape and texture of segmented lesions. Moreover, providing accurate lobe segmentation of COVID-19 patients would be challenging from typical low-dose and high pitch chest CT scans. In the current and previous studies^37,39,58^, radiomics features, as extracted from the entire lung (less challenging segmentation task for deep learning algorithms), were evaluated to provide fast and robust severity scoring in COVID-19 patients.

In this work, chest CT was used for assessment. At the same time, there are few studies on other modalities such as chest X-ray radiography in prognostication and outcome prediction evaluation of COVID-19 patients. For example, Bae and colleagues^60^ utilized radiomics features and modeled them on chest X-rays of 514 patients and found out that their radiomics- and deep learning-based model can accurately predict mortality and the need for mechanical ventilation in patients (AUCs = 0.93 and 0.90, respectively). Providing a severity score using chest X-rays is a valuable venue to explore. Yet, such work requires extensive comparisons with CT-based frameworks to assess the relative value of each modality for different tasks.

A number of radiomic features were selected with different IVs by two different algorithms. The 10Percentile, 90 Percentile and Mean from first-order features, which show the different percentile and Mean intensity within a region of interest were the selected features. The 10Percentile, 90 Percentile and Mean from first-order, despite max and min intensity, which are affected by noise, could be correlated with the involvement of the lung by infection as in severe cases, the infected lungs have high HU values. Other features selected by both algorithms was the Mean Absolute Deviation from first-order, achieving the highest IV in both algorithms. This feature is defined as the mean distance of image intensities from the mean value. As different stages of Covid-19 disease had different CT manifestations from no lesions, and medium to highly affected by infectious lesions, this feature could be correlated by stage of disease with different levels of infection demonstrated by the intensity of HUs. In addition to our study, these three features were selected by Yip et al.^39^ using the same datasets with different machine learning algorithms.

Zone Entropy (ZE) from GLSZM was another radiomics feature selected with high IV. This feature measures the randomness in distribution of the zones where a higher value indicates higher heterogeneity. Different stages of COVID-19 indicate different manifestations, including bilateral, multifocal, peripheral ground-glass opacities, consolidation, and crazy paving. These manifestations provide different textures where ZE could potentially be correlated with initial different heterogeneity generated by different stages. Dependence Variance (DV) from GLDM which measures the intensities variance had the highest IV in BRF algorithms. This feature could potentially be correlated with heterogeneities in different scores as severe cases had multiple types of lesions with high heterogeneity across the whole lung.

Gray Level Non-Uniformity Normalize from GLRLM was selected by both algorithms with high IV, which represents the spatial intensity changes in images. In severe COVID-19 cases, the lungs reveal more infections containing different types of manifestations resulting in high heterogeneity textures. In the case of high variability of intensity and high spatial change, such as high severe cases, the GLNUN feature value would be high. Gray Level Variance (GLV) from GLDM was another feature selected by MARS algorithm as high IV. GLDM calculates the coarseness of the texture whereas GLV feature measures the variance in dependence counts over intensities. This feature also quantifies the heterogeneity of regions of interest. In our study, this could be correlated with severe cases as the lung involves coarse textures of infection manifestation.

We presented the voxelwise feature map for three different features in different classes of severity. These features map visualize the different patterns of features across the different COVID-19 cases. In this study, we attempted to clinically interpret selected features, similar to previous studies^61–64^ with the aim to hypothetically correlate the selected features and biological phenomena in different classes of severity. We should note that multivariate analysis uses different information from the selected features and using only one feature as univariate analysis doesn’t yield high performance for scoring. The combination of these selected features could provide complementary information toward robust multiclass severity scoring modeling.

This study suffered from a few limitations, including the fact that our model was trained on single-center data. Further research should be conducted on large-scale and multi-centric data and patient images with multiple observers for improved training of the models and enhanced generalizability. In the current study, the developed models were compared only to previous studies. Further work should focus on the comparison of ML-based scoring models with conventional scoring approaches.

## Conclusion

We evaluated high-dimensional multinomial multiclass severity scoring of pneumonia using CT radiomic features and machine learning algorithms. We applied two feature selectors coupled to a classifier on a large cohort of COVID-19 patients. Our radiomics model was validated to depict accurate classification of patients according to multi-class pneumonia severity assessment criteria, highlighting the potential of this emerging paradigm in the assessment and management of COVID-19 patients. The selected radiomic features could be visualized to highlight the affected regions for better understanding of images, toward interpretable machine learning models. We proposed radiomics and machine learning-based high-dimensional multinomial multiclass severity scoring systems which could be potentially used in real clinical situations for severity assessment of COVID-19 patients. The proposed methods could be useful for highly affected (severe) COVID-19 patients management (ICU admission and treatment assessment).

## Abbreviations

CT: Computed tomography
COVID-19: Coronavirus disease 2019
AUC: Area under the receiver operating characteristic curve
CV: Cross-validation
BRF: Bagging random forest
FS: Feature selection
IV: Importance value
ML: Machine learning
GGO: Ground glass opacity
IBSI: The image biomarker standardization initiative
MARS: Multivariate adaptive regression splines
MLR: Multinomial logistic regression
RT-PCR: Reverse transcription polymerase chain reaction
GLCM: Gray-level co-occurrence matrix
GLSZM: Gray-level size-zone matrix
NGTDM: Neighbouring gray tone difference matrix
GLRLM: Gray-level run-length matrix
GLDM: Gray-level dependence matrix
